# Tinea Tonsurans

**Published:** 1894-04-21

**Authors:** H. W. Marett Tims

**Affiliations:** Lecturer on Biology and Comparative Anatomy at the Westminster Hospital Medical School, and Clinical Assistant at the Hospital for Skin Diseases, Blackfriars


### TINEA TONSURANS.

By H. W. Marett Tims, M.D., Lecturer on Biology and Comparative Anatomy at the Westminster Hospital Medical School, and Clinical Assistant at the Hospital for Skin Diseases, Blackfriars.

Ringworm of the scalp is one of the commonest, and at the same time one of the most troublsome affections of the skin. The great majority of the cases occur in children. It is stated that it is more common in fair-haired children, but this is very doubtful. Adults are very rarely attacked, but when they are the attack is much more amenable to treatment. It is eminently contagious, and when once it has broken out in an institution, as in a school, it is exceedingly difficult to eradicate, the two main reasons being the difficulty of stopping every source of infection among the children and the obstinacy of individual cases. Second attacks are rare, though relapses are very frequent.

*Causation.*—Ringworm is due to the presence of the Tricophyton tonsurans in the hair follicles. This fungus is probably a definite unit, and is not identical with many other fungi with which some authors wish to associate it. The spores* are of two sizes, large and small, called respectively macrospores and microspores. The mycelium and spores can very easily be demonstrated in an infected hair by the following simple process. It is put into a watch-glass containing ether, which dissolves the fat. A few minutes will suffice, but a more perfect specimen will be obtained by an immersion of some hours. It is then taken out and placed in liquor potassæ. The same remark as to the time required applies here also. The hair can then be placed on a slide and examined, or mounted in glycerine if required for preservation. The spores and mycelium are seen penetrating the hair fibres. Two views are held as to the mode of entrance in the hairs. One is that the fungus enters at the opening of the hair follicle, passes down between it and the hair root, and then grows up into the hair from below; the other, that the fungus passes directly into the hair at the surface, or a very little way down the follicle, and then extends upwards, but more particularly downwards, in the substance of the hair itself.

*Symptoms.*—A typical patch on the scalp presents the following characteristics. In an early stage there are a few inflammatory papules, which may give rise to some irritation. The hairs are brittle, and have lost their glossy appearance; the surface of the patch may be scaly, or covered with a mixture of scales and sebum. Very soon the place becomes bald, that is to say, there are no long hairs upon it. On careful examination the surface is found to be covered with a number of short stumps of hair, broken off abruptly at the free extremity. These are invisible to the naked eye, but can be clearly distinguished with an ordinary pocket lens. The stumps can, with a little practice, be readily pulled out; but, owing to their brittleness, are very apt to break out. These broken hairs are very characteristic of ringworm. If examined under the microscope, after being prepared as described above, the fungus will be seen as well as the abrupt and jagged end of the hair. This is, of course, conclusive evidence. Such a typical patch may not always be seen. The irritation caused may lead to so much scratching that the surface may be covered with impetiginous crusts, which must be removed before the characteristic stumps can be seen. Or, they may be hidden by a thick layer of scales and sebum, which may mat down the neighbouring hairs. Nevertheless, by lifting up some of the scales, or by the application of a little liquor potassæ, they can generally be made out. The application of ungt. ac. boracis for a few days will clean up the patch and make the diagnosis easy. Another practical point is the application of chloroform, as suggested by Sir

* For detailed account *vide British Medical Journal*, proceedings of Dermatol. Section at annual meeting at Newcastle in August last.

Dyce Duckworth; this turns the affected hairs almost white. This test, though valuable, is not infallible. *Kerion* is a pustular folliculitis, which is occasionally set up by the presence of the fungus, but more often by the application of irritation substances. The inflammation gives rise to a well-marked reddened area, with numerous pustules; the whole having a boggy or fluctuating feel. A separate condition has been described as bald tinea tonsurans, which gives rise to patches almost identical with alopecia areata, and with which it will be considered in a future paper. *Tinia circinata,* ringworm of the body, requires no special description. It is easily recognised, and, as a rule, yields readily to treatment. *Differential diagnosis* lies mainly between ringworm, seborrhœa, and psoriasis. (i) *Seborrhœa* is more diffuse; it may be all over the scalp. There is a general thinning of the hair, but no marked patches of baldness and no characteristic stumps. (ii) *Psoriasis* of the head is usually associated with patches on the body in its typical situations. Hence, in all cases of doubt, the elbows and knees should be examined. If confined to the head, there is more scaliness, but no loss or breaking short of the hairs. Lastly, I would urge that in all cases of doubt, in all cases where a very decided opinion has to be given, and always before discharging the patient as cured, a microscopic examination should be made. In private practice I would advise that this should be done in every case, as there is probably no skin affection about which so many erroneous diagnoses are made, and which could be so easily prevented and professional reputation saved.

*Treatment* is mainly in two directions, either to kill the fungus or to render the soil unfit for its development. The difficulty in the former plan is to get the medicament far enough down into the hair follicle to reach. Two points are, I think, essential as a matter of routine. First, the hat should always be lined with calico, or even paper, which can be taken out and burnt and renewed two or three times a week. If this be not done the hat will remain a constant source of infection. The second point is to wash the scalp all over every night with some parasiticide lotion as of hydrarg. perchlor. (gr. i. to ℥i.), and thus prevent the occurrence of fresh sites. If the patches be numerous the whole head should be shaved; if few in number, it will only be necessary to cut the hair short around each one. With regard to the application to be adopted it is very difficult to speak with any certainty. Very many have been advocated, and each one seems to do good in certain instances, but fails in other. Indeed, we now and again meet with cases that seem to resist all efforts at treatment, and go on steadily until the age of puberty, at which time they not infrequently disappear. The method of treatment I have found as successful as any is the application of the following ointment:—℞ ac. carbol, ac. salicyl āā. ʒ ss., vaselini ℥i. This should be well rubbed in night and morning. The head should be well washed two or three times a week with with warm soap and water. If any reliable person is available, the diseased hairs should be systematically epilated. If this treatment be not successful, I then use chyrsarobin, beginning with a strength of 10 grains to the ounce, and gradually increasing up to 20 or even 30 grains, care being taken to prevent its getting into the patient's eyes. This frequently effects a cure; but some cases seem to improve up to a certain point and then stop. In such cases the further application of oleate of mercury ointment (5 per cent. or 10 per cent.) will generally be sufficient. I have sometimes, in early cases, had good results with boracic acid, using Dr. Cavafy's formula, which is boracic acid, 20 grains; ether, a drachm; spt. vini. rectif., one ounce. In chronic cases, Coster's paste, or Mr. Morrant Baker's modification of this are very useful. Whichever of all these is adopted, it must be thoroughly rubbed in and not merely painted on. Some American physicians have told me that they place great reliance on the application of a solution of hydrargperchlor. in ether (gr. i. to ℥i.). I have tried it, and do not consider that it possesses any advantage over the method I have just given, while the danger from absorption, as evidenced by recorded cases, more than counterbalances the questionable advantage. Where any impetiginous crusts are present, they should first be removed by means of poultices; and any thick layer of scales by ungt. ac. boracis.

The importance of having separate brushes, combs, towels, &c., for the patient's use is too well known and recognised to require special mention. Want of space prevents me from going into the number of other substances which have proved of use, such as pyrogallic acid, which is, I believe, much in vogue in Vienna, salicylic acid, boro-glycerine, sulphur, &c. I have merely indicated the line of treatment which I usually adopt.

